# ‘HepCheck Dublin’: an intensified hepatitis C screening programme in a homeless population demonstrates the need for alternative models of care

**DOI:** 10.1186/s12879-019-3748-2

**Published:** 2019-02-07

**Authors:** John S. Lambert, Ross Murtagh, Dee Menezes, Austin O’Carroll, Carol Murphy, Walter Cullen, Tina McHugh, Gordana Avramovic, Willard Tinago, Marie Claire Van Hout

**Affiliations:** 1 Infectious Diseases Department, Mater Misericordiae University Hospital, Dublin, Ireland; 2 UCD School of Medicine, University College Dublin, Dublin, Ireland; 3 University College London, London, UK; 4 Safetynet Primary Care Network, Dublin, Ireland; 5 Mater Misericordiae University Hospital, Dublin, Ireland; 6 Public Health Institute, Liverpool John Moores University, Liverpool, L32ET UK; 7 Department of Infectious Diseases, Catherine McAuley Centre, 21 Nelson Street, Phibsborough, Dublin 7, Dublin Ireland

**Keywords:** Hepatitis C virus, HCV, homeless

## Abstract

**Background:**

Hepatitis C virus (HCV) is one of the main causes of chronic liver disease worldwide. Prevalence of HCV in homeless populations ranges from 3.9 to 36.2%. The HepCheck study sought to investigate and establish the characterisation of HCV burden among individuals who attended an intensified screening programme for HCV in homeless services in Dublin, Ireland.

**Methods:**

The HepCheck study was conducted as part of a larger European wide initiative called HepCare Europe. The study consisted of three phases; 1) all subjects completed a short survey and were offered a rapid oral HCV test; 2) a convenience sample of HCV positive participants from phase 1 were selected to complete a survey on health and social risk factors and 3) subjects were tracked along the referral pathway to identify whether they were referred to a specialist clinic, attended the specialist clinic, were assessed for cirrhosis by transient elastography (Fibroscan) and were treated for HCV.

**Results:**

Five hundred ninety-seven individuals were offered HCV screening, 73% were male and 63% reported having had a previous HCV screening. We screened 538 (90%) of those offered screening, with 37% testing positive. Among those who tested positive, 112 (56%) were ‘new positives’ and 44% were ‘known positives’. Undiagnosed HCV was prevalent in 19% of the study sample. Active past 30-day drug use was common, along with attendance for drug treatment. Unstable accommodation was the most common barrier to attending specialist appointments and accessing treatment. Depression and anxiety, dental problems and respiratory conditions were common reported health problems. Forty-six subjects were referred to specialised services and two subjects completed HCV treatment.

**Conclusions:**

This study demonstrates that the current hospital-based model of care is inadequate in addressing the specific needs of a homeless population and emphasises the need for a community-based treatment approach. Findings are intended to inform HepCare Europe in their development of a community-based model of care in order to engage with homeless individuals with multiple co-morbidities including substance abuse, who are affected by or infected with HCV.

## Introduction

Hepatitis C virus (HCV) is one of the main causes of chronic liver disease worldwide [1]. The long-term impact of HCV infection is highly variable, from minimal changes to extensive fibrosis and cirrhosis with or without hepatocellular carcinoma (HCC) [2, 3]. Acute infection is asymptomatic in 60–70% of cases, meaning that many do not become aware that they are HCV positive until decades after initial infection, after progression of the disease and emergence of sequelae [4]. Effective diagnosis and treatment is heavily reliant on screening of at-risk individuals [5]. The number of chronically infected persons worldwide is estimated to be approximately 71 million [6]. The implementation of extended criteria for screening of HCV is currently the subject of major debate among different stakeholders [7].

A 2012 systematic review and meta-analysis of HCV in homeless populations found prevalence ranging from 3.9 to 36.2% [8]. Homelessness is associated with an increased prevalence of risk factors for HCV such as injecting drug use (IDU) [9, 10]. The link between homelessness and poor health is well established [11–13]. Homeless individuals generally delay going to a health service and so when they eventually do present, it is generally late on in their condition and can often have become so severe that they require hospitalisation. Preventing the transmission of blood borne viruses (BBV) such as HCV is therefore a key responsibility of community health services and their partners in homeless services [8, 14–17]. High levels of alcohol use, as well as diabetes and metabolic syndrome suggest probable higher rates of fatty liver disease among this population [9, 18].

In Ireland, where our study took place, it is estimated that 20,000–50,000 people are chronically infected with HCV [19]. Of the six HCV genotypes, Genotype 1 and 3 are most common in Ireland. National data on HCV infection in Ireland amongst drug users is restricted to certain treatment sites and prisons [20]. Keegan et al. (2017) in their study reported that prevalence of HCV infection among drug users was 63.6% with no significant gender difference and with prevalence lower in younger adults. General prevalence estimates within the drug user populations range from 54% [21] to 84% [22]. In terms of homeless adults in Dublin, in 2005 the rate of HCV infection was estimated to be 36% [23]. A 2015 study of people who are homeless/at risk of homelessness receiving free primary healthcare in Dublin reported a HCV prevalence of 23% [16]. Information on the extent of chronic liver disease amongst homeless individuals in Ireland is however limited. There is substandard uptake in HCV assessment and treatment among people who inject drugs (PWID) in Ireland [24].

The Irish Health Service Executive (HSE) funded Safetynet Primary Care service is a network of general practitioners and nurses providing primary care to homeless and other marginalised groups. This multifaceted service includes the offering of treatment in their own environment to homeless people who are addicted to heroin, thus removing many of the barriers that result in their exclusion from treatment. It provides a novel alternative to the centralised clinic approach, which was the only option for homeless people prior to the project’s establishment. In Dublin, homeless heroin dependant patients can access methadone maintenance treatment through Safetynet services (as well as HSE addiction centres). Our study sought to evaluate an intensified screening programme for HCV in homeless individuals attending Primary Care services in Dublin, Ireland, and in doing so investigate and establish the characterisation of HCV burden among this cohort.

## Methods

HepCheck Dublin was undertaken as part of a larger European wide initiative called HepCare Europe. HepCare Europe is an EU-supported service innovation project and feasibility study at four European sites (Dublin, London, Seville and Bucharest) to develop, implement and evaluate interventions to enhance identification and treatment of HCV among vulnerable populations [25]. The project focuses on providing an ‘integrated care’ model for HCV treatment based on collaboration between primary and specialist care practitioners to allow for more efficient use of limited specialist resources. The HepCheck Dublin project looks specifically at screening individuals using rapid HCV testing and tracking results and pathways to care outcomes.

This study used a cohort design. The target population were homeless individuals accessing the Safetynet Primary Care services in Dublin. Individuals using homeless services and hostels (Merchant’s Quay Ireland (MQI), Capuchin Day Centre, Ana Liffey Drug Project (ALDP), Salvation Army, De Paul Ireland, Crosscare, the Dublin Simon Community and the Safetynet mobile health unit) were informed of screening for HCV in Safetynet clinics and encouraged to attend. Individuals who reported that they had already been diagnosed as having HCV were advised to attend a safetynet clinic for assessment and referral for treatment if appropriate. Screening took place in 11 Safetynet affiliated GP practices, as well as Safetynet in-reach locations such as coffee shops, needle exchanges, hostels, drop in centres, and the Safetynet mobile health unit.

Inclusion criteria were as follows: 1) attended Safetynet services during the study period, 2) age > 18, and 3) ability to provide informed consent. Exclusion criterion was defined as inability to provide informed consent. Subjects were given an information sheet on the study and provided written consent. Subjects were recruited over a 19-month period, from December 2014 to July 2016. As conventional probability methods, such as simple or stratified random sampling, are often not appropriate for populations of injecting drug users [26], targeted sampling, a non-probability sampling framework, was used to identify subjects on whom data was collected [27]. Subjects received no financial incentive for participation in the study.

The HCV process audit consisted of 3 phases;

- In Phase One, during an intensified community HCV screening program, all subjects attending services were invited to complete a short questionnaire and offered blood test or rapid oral HCV test, and if found positive, participation in Phase 3 of the study. OraQuick® oral swab was used as a point of care test for HCV antibodies. Each subject completed a questionnaire which was administered by a research assistant who accompanied the nurse. This questionnaire collected information on the number of individuals offered HCV screening, number of individuals screened, proportion of individuals offered screening who already had been diagnosed as having HCV, the treatment status of individuals already diagnosed with HCV (cured/treated but not cured/awaiting treatment/defaulted from follow-up/never referred to specialist treatment), the proportion of individuals with positive HCV antibody (HCV Ab+) on screening, and the number of HCV Ab+ individuals (either new or previously diagnosed) attending for HCV assessment. Demographic information was also collected regarding age, gender, ethnicity, injecting drug use, alcohol use, accommodation status and keyworker involvement. The keyworker is a member of a multidisciplinary team who is tasked with coordinating service user’s support plan.

As the treatment pathway in Ireland is restricted to hospital based care HCV+ patients were referred to Mater Misericordiae University Hospital for specialist care.

- In Phase Two, a convenience sample of participants who in Phase 1 reported previously testing HCV positive (i.e. known positive) were administered an in-depth questionnaire by a researcher during screening clinics. The questionnaire comprised 79 closed and open-ended questions. Questions asked explored health and social risk factors, including: homelessness experience, health status, health service usage, co-morbidities, when diagnosis was made and the steps that were taken post positive HCV Ab test. The purpose of this questionnaire was to identify barriers to follow-up.
- Lastly, in Phase 3, subjects testing positive in Phase 1 were tracked along the referral pathway to identify whether they were referred to a specialist clinic, attended the specialist clinic, were assessed for cirrhosis by Fibroscan and were treated for HCV. Where possible, a key worker was involved in linking the subject to specialist care. Subjects were given a minimum of 3 appointments for a specialist clinic. Subjects were eligible for treatment with direct-acting antivirals if they had a Fibroscan score of > 12.5 kPa as per national protocols (at time of testing).

The data were analysed using SPSS v24. Descriptive statistics (frequencies, percentages, median and interquartile range (IQR)) were used to summarise subjects’ characteristics. In analysing Phase Three data we used unadjusted negative binomial regression (NBR) models to examine the factors associated with the number of specialist care visits. The following explanatory variables were considered; age, gender, alcohol and drug use, key worker involvement and accommodation status. This model has been shown to have better fit than Poisson regression models by accounting for statistical overdispersion and excess zeros (individuals with no specialist care visits) and yields a more conservative result. The parameter estimates from the NBR models were converted to incidence rate ratios (IRRs) by exponentiating the regression coefficients, and 95% confidence intervals were estimated. Incidence rate ratio above 1.0 implies higher number of specialist care visit and IRR below 1.0 implies lower number of specialist care visits.

## Results

### Phase one- screening and survey

A total of 597 subjects were offered HCV screening and recruited into the study, their median (IQR) age was 36 (29–43) years and 438 (73%) were male. Self-reported information regarding IV drug use was complete on 157 subjects, with 54% reporting current IV drug use. Characteristics of the sample recruited into the HepCheck Dublin study are presented in Table 1.

**Table 1:** Characteristics of the sample recruited into the HepCheck Dublin study

	*N* = 597
Age - years (median (IQR))	36 (29–43)
Gender *n* (%)	
Male	438 (73%)
Female	159 (27%)
^a^Ethnicity (*n* = 286)	
Irish	281 (98%)
Other European	5 (2%)
^a^Temporary emergency accommodation^b^ (*n* = 247)	
Yes	177 (72%)
No	70 (28%)
^a^Key worker involvement (*n* = 194)	
Yes	143 (74%)
No	51 (26%)
^a^Injection drug use (*n* = 157)	
Current^b^	84 (54%)
Past	68 (43%)
Never	5 (3%)
^a^Alcohol use^c^ (*n* = 193)	
Yes	72 (37%)
No	121 (63%)

Note ethnicity missing 311 responses, temporary emergency accommodation missing 350 responses, key worker missing 403 responses, injecting drug use missing 440 responses and alcohol use missing 404 responses

^a^ Proportions based on complete cases with response available on the characteristic

IQR: interquartile range

^b^ Secured for 6 months

^b^ In past month

^c^ Self-reported problematic alcohol use

Out of 597 recruited subjects, only 353 provided information on previous HCV screening. Of the 353, 223 (63%) reported having been screened previously for HCV of whom 100 (45%) indicated the results being positive, 78 (35%) negative and 45 (20%) were unsure (Table 2). Almost half of those with reported previous HCV screening indicated the screening having been conducted more than 2 years ago.

**Table 2:** Proportion of subjects with previous HCV screening and details of previous screening

	*n* (%)
Number who responded	353/597 (59%)
Previously screened (*n* = 353)	
Yes	223 (63%)
No	130 (37%)
If previously screened, when? (*n* = 223)	
6 months	47 (21%)
1 year	52 (23%)
2+ years	108 (48%)
Unknown	16 (7%)
Results of those previously screened (n = 223)	
Positive	100 (45%)
Negative	78 (35%)
Unsure	45 (20%)
Previously offered treatment	34 (34%)

During Phase 1, we conducted HCV Ab tests in 538/597 subjects (59 opted out). Of those who received an HCV Ab test, 199 (37%) tested positive, 308 (57%) negative and 31 (6%) were recorded as no result. Of the 199 testing positive, 56% (112) were “new” positives, in that they did not report having had a previous positive HCV Ab test, indicating an overall undiagnosed HCV prevalence of 19% (112/597). The remainder of those testing positive were known positives (Table 3).

**Table 3:** Phase 1 HepCheck Screening Intervention results

	*n* (%)
Received HCV Ab test (n = 597)	
Yes	538 (90%)
No	59 (10%)
HCV Ab test results (*n* = 538)	
Positive	199 (37%)
Negative	308 (57%)
No result	31 (6%)
Classification of HCV Ab positives (*n* = 199)	
New positives	112 (56%)
Known positives	87 (44%)
HCV Ag testing on Ab positives (*n* = 112)	
Yes	13 (12%)
No	99 (88%)
HCV Ag results (*n* = 13)	
Positive	12 (92%)
Negative	0 (0%)
Unknown	1 (8%)

*HCV* Hepatitis C Virus, *Ab* antibody, *Ag* antigen

### Phase two- in-depth questionnaire

**Fig. 1:**
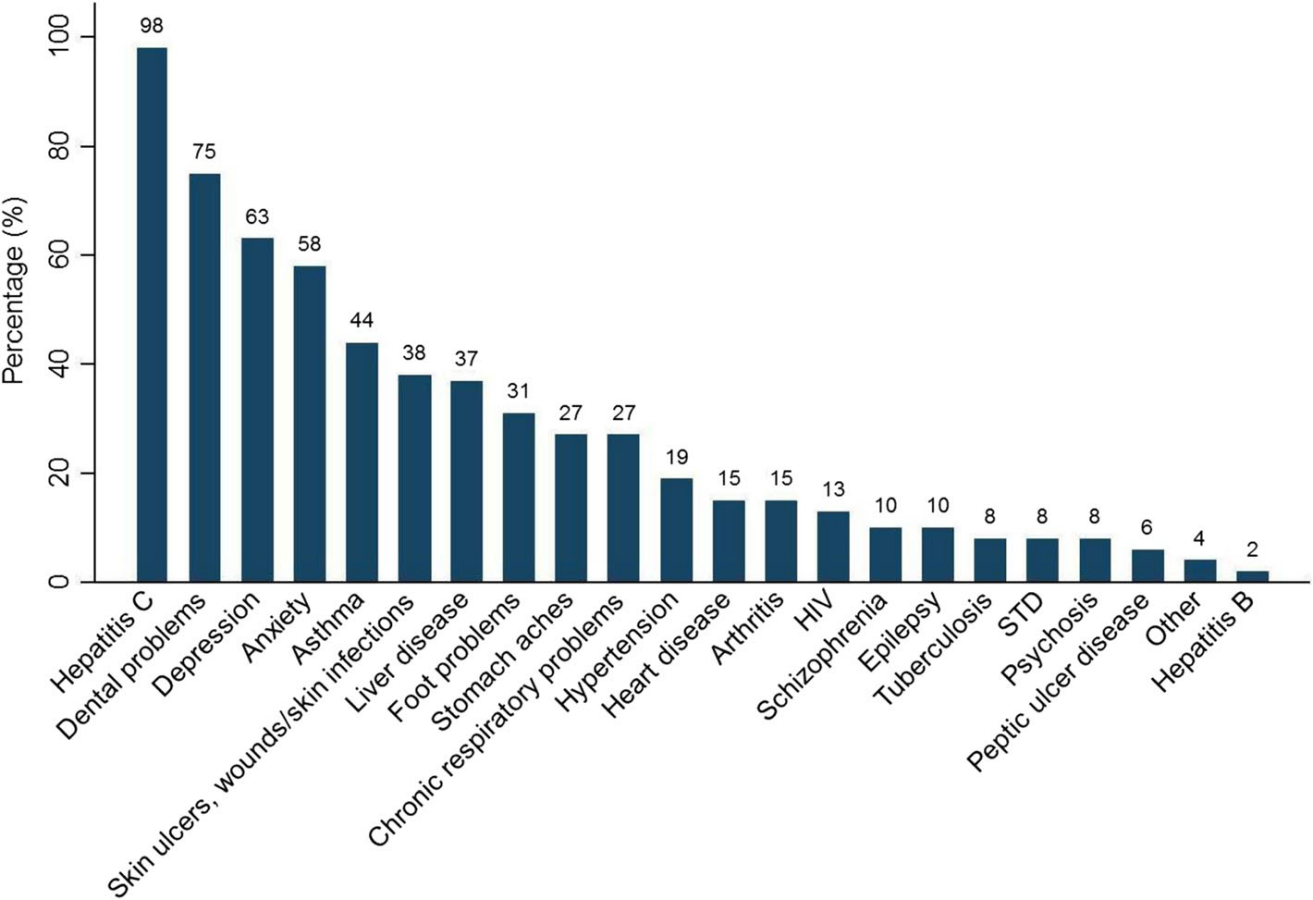
‘Distribution of morbidities in Phase 2 subjects who reported previous positive HCV test in Phase 1’

Forty eight subjects who reported previously testing positive for HCV in Phase 1 were willing to participate in Phase 2 and complete a researcher administered questionnaire. The majority were male (69%), and 78% were currently living in a hostel. The remaining were ‘sofa surfing’, sleeping rough or staying with friends. 85% were homeless for longer than 1 year. The average time period of homelessness was 6.2 years, with a range of 2 months to 20 years. The most common reasons for homelessness were co-morbidities such as alcohol and/or drugs, and for some, this was combined with family/relationship problems and mental health problems. Forty-two percent reported seeing a GP once per week for unspecified reasons. In order to assess morbidity, respondents were read a list of physical and mental health problems and asked ‘Have you ever been told by a doctor that you have one of the following?’ Table 4 illustrates their health status. Depression and anxiety, dental problems and respiratory conditions were common reported health problems (Fig. 1). 69% reported use of drugs in the past 30 days, with 45% ever sharing needles and 73% currently attending a drug treatment centre.

**Table 4:** Health Status in Phase 2 subjects who reported previous positive HCV test in Phase 1

	*n* = 48
Self-rated health	
Good to excellent	14 (29%)
Fair to poor	33 (69%)
Health worse than one year ago	21 (44%)
Service usage	
No medical card	18 (38%)
Not registered with a GP	14 (29%)
Attended ED in past six months	31 (65%)
Medications	
On prescription medication	37 (77%)
Drug use and addiction	
Illicit drug use in the past 30 days	
Heroin	34 (71%)
Benzodiazepines	23 (48%)
Crack cocaine	11 (23%)
Other	10 (21%)
Age of first IV drug use (*N* = 44)	
< 12 years	1 (2%)
12–14 years	6 (14%)
14–16 years	7 (16%)
16–18 years	5 (11%)
18–21 years	8 (18%)
21 and over	17 (39%)
Ever shared needles	20 (42%)
Currently attending drug treatment centre	32 (67%)

*ED* Emergency Department, *IV* Intravenous, *GP* General Practitioner

When asked about the status of their HCV infection, 77% disclosed that they were unaware of the current status of their infection, 9% reported that they had cleared the infection / attained sustained viral response (SVR), and 6% had ‘active’ infection. Regarding engagement with follow-up, 63% (*n* = 30) had been previously referred to specialist care, and among these 60% (*n* = 18) had attended at least one appointment. 50% (*n* = 9) of those who had attended specialist care previously had stopped attending (Table 5). When asked about their HCV healthcare pathway, unstable accommodation was reported to be the most common barrier to attending specialist appointments and accessing treatment. The most common other reasons for non-attendance were active drug use, being in prison, fear of side effects of treatment and forgetfulness.

**Table 5:** Historic HCV care processes in Phase 2 subjects who reported previous positive HCV test in Phase 1

	n = 48
Tested for Hepatitis C	
Positive diagnosis	48 (100%)
Status of infection	
Cleared/SVR	4 (8%)
Chronic	6 (13%)
Unknown	38 (79%)
Further testing	
Ultrasound	18 (38%)
Liver biopsy	7 (15%)
Fibroscan	2 (4%)
Referred to specialist care	30 (63%)
Attended specialist care	18 (38%)
Stopped attending	9 (19%)
Offered treatment	7 (15%)
Not treated, but would like to be treated now	34 (71%)

*SVR* Sustained virologic response

### Phase three- referral and outcome tracking

**Fig. 2:**
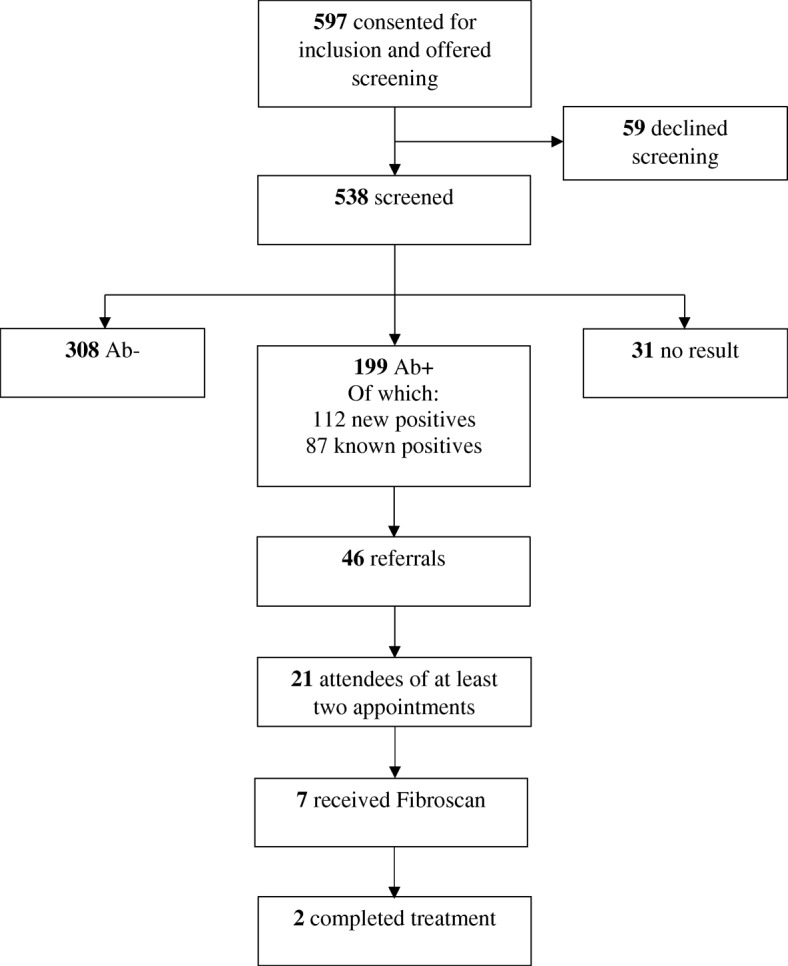
‘Screening flowchart’

Following a positive HCV Ab test, 46 subjects were referred to specialist care, of which 21 attended at least two appointments. Seven subjects received a Fibroscan or ultrasound. At time of writing, two subjects had completed treatment. See Fig. 2.

Table 6 below shows factors associated with specialist care visits. In the unadjusted NBRs for Phase 3, no associations were observed between the number of specialist care visits and the examined factors: age, gender, drug or alcohol use, stable accommodation status and key worker involvement.

**Table 6:** Unadjusted negative binomial regression for factors associated with specialist care attendance

Characteristics	IRR	95% CI	*P*-Value
Age (per 5-year increment)	0.92	0.78–1.10	0.37
Gender			
Female	Referent		
Male	1.45	0.72–2.89	0.28
Drug/alcohol use			
Yes	0.82	0.45–1.49	0.51
No	Referent		
Accommodation for six months			
Yes	1.69	0.64–4.47	0.29
No	Referent		
Key Worker			
Yes	1.48	0.57–3.82	0.41
No	Referent		

*IRR* incidence rate ratio, *CI* confidence interval

## Discussion

This is the first study in Ireland to specifically target and characterise homeless individuals and their prevalence of HCV and experiences of the HCV care pathway. The study presents a unique Irish profile of HCV burden among homeless patients accessing primary care services in Dublin, Ireland, and illustrates the complexities around provision of an intensified screening programme for HCV for homeless populations. The strengths of the study include the selection of a particularly hard to reach group of homeless individuals in Dublin, who are a HCV risk population who need to be studied and intervened with in order to better understand how to improve their experiences of HCV assessment and treatment.

The study highlights the complexities around provision of HCV cascade of care to homeless individuals living in Dublin. Not having stable accommodation is a major barrier to screening and treatment retention. Studies elsewhere in Europe have indicated how structural factors impede HCV screening and treatment access, with key barriers centring on social stigma, housing, criminalisation, healthcare systems, and gender [28].

As highlighted previously, hospital-based appointments are the only model of care available in Ireland. This study demonstrates that standard referral methods are inadequate in engaging this population. The current referral process exacerbates barriers to treatment engagement. Due to the transient nature of the cohort there are difficulties in contacting and sending reminders for appointments. To compound this further, not all participants had access to a keyworker to facilitate the referral process.

The opportunistic community-based screening uncovered concerning numbers of HCV Ab positive individuals in those accessing SafetyNet services, with 37% of individuals testing positive for HCV antibodies. Of note is that over half were ‘new positives’ and just under half were ‘known positives’, which underscores the need to enhance patient engagement with the HCV cascade of care, and to completion. Studies in Dublin have pointed to the issues around other disease morbidities in this population where symptoms of alcohol or drug withdrawals may mask HCV symptoms [24]. This highlights that symptom-based testing for HCV is futile, further emphasising the necessity for broad risk-based and network-based strategies in effectively identifying cases [5, 29].

The study supports Crowley et al. (2017) by emphasising the need to consistently target homeless individuals, particularly those who are actively using or have a history of injecting drug use, for HCV screening and treatment. Marginalised injecting drug users, especially those who are without stable accommodation are a hard to reach group and are less likely to access health care due to fear of discrimination, stigma, low health literacy, and health professional misunderstanding of their needs [20, 30–32]. Homeless patient characteristics in our study are equally complex in terms of physical and mental health problems such as continued drug/alcohol use, being on drug treatment, and experiencing forgetfulness, depression and anxiety, respiratory and dental conditions, all of which confound the HCV treatment care pathway. Reasons for non-attendance of HCV care such as active drug use, being incarcerated, fear of HCV treatment side effects and forgetfulness are similar to those reported by Crowley et al. (2017) in their study of Irish patient characteristics and barriers to Hepatitis C treatment in patients on opioid substitution treatment (OST) attending a community-based clinic where Fibroscans were performed.

Scaling up of HCV prevention, screening and treatment to address HCV transmission among the homeless, particularly those injecting drugs within existing community-based infrastructures and primary care are warranted [20, 31–33]. Of note is that among Phase 2 participants, 42% saw a GP once a week. This suggests a potential for high engagement within community-based primary care settings. A recent study by Barocas et al. (2017) describes excellent responses to community-based HCV treatment within a primary care program in a cohort of homeless and marginally housed adults, demonstrating that despite barriers and competing priorities, outcomes similar to those of clinical trials and other cohorts are possible for this traditionally difficult to treat group [34]. Similarly, Grebely et al. (2017) describes an effective implementation of point-of-care HCV RNA testing in people attending drug health and homelessness services in Australia, showing potential for improved screening, linkage to care and treatment retention [35].

We recognise that whilst community-based screening intervention can determine the prevalence of HCV in homeless populations, referrals to / attendance at secondary care remains a challenge for this cohort. Psychosocial factors are at the core of why patients do not attend secondary care for HCV management and in this study, the interplay between addiction, mental health and homelessness were especially problematic. With this in mind, community-based health services such as Safetynet are ideally placed to support HCV screening and facilitate vulnerable individuals in accessing the treatment and care pathway. This study demonstrates that the current hospital-based model of care is inadequate in addressing the specific needs of this population, reflected in only 2 of the 199 individuals testing positive accessing treatment and cure. This emphasises the need for a community-based treatment approach.

### Limitations

A notable limitation of this study is missing data. Participating services were highly burdened, with a large volume of people accessing primary care and although there was high uptake of screening, a high number of questionnaires were incomplete as immediate health concerns took precedence in the limited consultation time. This meant that we were not as fully able to explore patient characteristics and barriers to access.

It is important to note that the HepCheck study was carried out during a homelessness crisis in Dublin. Sourcing accommodation on a nightly basis was an unfortunate reality for many of the participants in this study.

## Conclusion

The homeless population remain a high risk and high prevalence population for HCV infection [8–10]. Health literacy, screening uptake and treatment engagement are challenging given the complexities of this patient cohort. Findings from this study represent a first step in Ireland to understanding the needs of homeless individuals and are intended to inform HepCare Europe in their development of a community-based model of care in order to engage with homeless individuals who are infected with or affected by HCV.

## Abbreviations

Ab: Antibody
Ag: Antigen
BBV: Blood borne virus
ED: Emergency department
GP: General practitioner
HCC: Hepatocellular carcinoma
HCV: Hepatitis C virus
HIV: Human immunodeficiency virus
HSE: Health Service Executive
IDU: Injecting drug use
IQR: Interquartile range
IRR: Incidence rate ratios
IV: Intravenous
NBR: Negative binomial regression
PWID: People who inject drugs
STD: Sexually transmitted disease
SVR: Sustained virologic response
